# Intrinsic Capacitances and Inductances of Quantum Hall Effect Devices

**DOI:** 10.6028/jres.101.071

**Published:** 1996

**Authors:** M. E. Cage, A. Jeffery

**Affiliations:** National Institute of Standards and Technology, Gaithersburg, MD 20899-0001

**Keywords:** impedance standard, internal capacitance, intrinsic impedance, kinetic inductance, magnetic inductance, quantum Hall effect, two-dimensional electron gas

## Abstract

Analytic solutions are obtained for the internal capacitances, kinetic inductances, and magnetic inductances of quantum Hall effect devices to investigate whether or not the quantized Hall resistance is the only intrinsic impedance of importance in measurements of the ac quantum Hall effect. The internal capacitances and inductances are obtained by using the results of Cage and Lavine, who determined the current and potential distributions across the widths of quantum Hall effect devices. These intrinsic capacitances and inductances produce small out-of-phase impedance corrections to the in-phase quantized Hall resistance and to the in-phase longitudinal resistance.

## 1. Introduction

The integer quantum Hall effect [1–3] requires a fully quantized two-dimensional electron gas (2DEG). At low currents there is negligible dissipation within the interior of the 2DEG in the quantum Hall plateau regions of high-quality devices, and the longitudinal resistance *R_x_* along the device is very small. Within these plateau regions the quantized Hall resistance *R*_H_ across the device has the value *R*_H_(*i*) = *h*/(*e*^2^*i*) for the *i*th plateau, where *h* is the Planck constant, *e* is the elementary charge, and *i* is an integer. We assume here that the quantity *iR*_H_(*i*) has the value of the von Klitzing constant *R*_K_ = 25 812.807 Ω.

The quantum Hall effect has been used to realize a device-independent resistance standard of high accuracy for dc currents [4, 5] and for very low frequency currents below 4 Hz [6]. An ac quantum Hall effect impedance standard is now being developed for alternating currents having frequencies of order 10^3^ Hz and angular frequencies of order 10^4^ rad/s [7–10].

Impedances are complex quantities, and can therefore have both real and imaginary components. If it is to be a useful absolute or intrinsic ac resistance standard, the impedance *across* the device must be dominated by the real component, which is the quantized Hall resistance *R*_H_(*i*), and the impedance *along* the device must be small and again dominated by the real component, which is the longitudinal resistance *R_x_*. The imaginary components (the *internal* or intrinsic impedances due to capacitances and inductances of the quantum Hall effect device itself) must provide small contributions in order to avoid a significant out-of-phase or quadrature signal.

There are, of course, *external* capacitances and inductances in the sample probe arising from the sample holder, bonding wires, and coaxial cables. We do not consider the external impedances here, but they must also be accounted for. Signals that are in-phase with *R*_H_(*i*) and *R_x_*, due to products of external and internal capacitances and inductances, can also be present. These second-order effects must also be small if the device is to be a useful intrinsic standard.

Seppa, Satrapinski, Varpula, and Saari [11] have estimated that the kinetic inductance contribution to the imaginary (*j*) component of the impedance can be of order 1 Ω, which is very large compared with *R_x_*. We therefore calculate the internal kinetic inductance, as well as the magnetic inductance, along the device to see if the imaginary components are indeed significant compared with the longitudinal resistance *R_x_*. We also calculate the internal capacitance across quantum Hall effect devices to see if it provides a significant out-of-phase contribution to the quantized Hall resistance *R*_H_(*i*). (We will find that there are no contributions from the internal capacitance along the device or from the kinetic and magnetic inductances across the device.)

The calculations have analytic solutions which utilize results from the work of Cage and Lavine [12, 13], who calculated potential, electric field, current, and current density distributions across the 400 μm width of a quantum Hall effect device for applied currents between 0 μA and 225 μA. The potential distributions of Cage and Lavine [12] are in excellent agreement with the experimental measurements of Fontein et al. [14], who used a laser beam and the electro-optic Pockels effect as a contactless probe of the 2DEG.

## 2. Potential and Current Distributions

A current *I*_SD_, externally applied between the source S and the drain D of a mesa-etched quantum Hall effect device, is confined to flow with a current sheet density *J*_t_ within the 2DEG layer of the device. This applied current induces a potential distribution across the device in the presence of a perpendicular external magnetic flux density *B*. The total potential difference across the device width *w* is the quantum Hall voltage *V*_H_(*i*) = *R*_H_(*i*)*I*_SD_.

Cage and Lavine [12] have calculated the potential distributions for applied currents between 0 μA and 225 μA. Their potential distributions are composed of parabolically shaped confining potentials (due to homogeneous charge-depletion regions) located on either side of the device, and a logarithmically shaped charge-redistribution potential (due to the Lorentz force exerted on the conducting electrons in the 2DEG causing deviations from the average surface charge density) extending across the device interior.

**Fig. 1:**
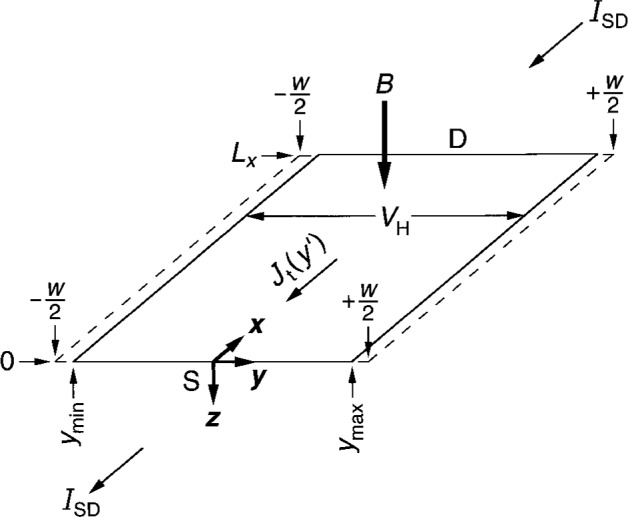
A 2DEG conducting sheet, with the origin of the coordinate system located at the source S, halfway across the device width *w*. The device length is *L_x_*, and D is the drain. Conducting electrons extend across the device from *y*_min_ to *y*_max_. *B* is the external magnetic flux density, *V*_H_ the quantum Hall voltage, *I*_SD_ the applied current, and *J*_t_(*y*′) the total current density at point *x*′, *y*′, 0 in the 2DEG.

Figure 1 is a schematic drawing of the 2DEG, with the origin of the coordinate system located at the source S and halfway across the 2DEG. On a quantum Hall plateau, the conducting electrons within the 2DEG occupy all the allowed states of filled Landau levels. The spatial extent of the conducting electrons lies within the device width *w*, and between the coordinates *y*_min_ and *y*_max_. We are interested in effects within the device interior, so we will neglect the fact that the applied current *I*_SD_ enters and exits opposite corners of the device. The electrons are therefore flowing only in the positive *x* direction at this instant of time. This corresponds to a current of positive charges moving in the negative *x* direction. The potential on the left hand side of the device is positive relative to the potential on the right hand side for these particular current and magnetic flux density directions.

### 2.1 Results of Cage and Lavine

For convenience, we repeat those results of Cage and Lavine [12] that are used as the starting points of our calculations. The equations for the confining potential *V*_c_ at any point *y*′ across the 2DEG are

$$V_{c}\left(y\prime \right)=-a{\left(y\prime -\lambda \right)}^{2}\;\text{for}\;\lambda \leq y\prime \leq \frac{w}{2}$$ (1a)

$$V_{c}\left(y\prime \right)=0\;\text{for}-\lambda <y\prime <\lambda$$ (1b)

$$V_{c}\left(y\prime \right)=-a{\left(y\prime +\lambda \right)}^{2}\;\text{for}-\frac{w}{2}\leq -y\prime \leq -\lambda ,$$ (1c)

where

$$\lambda =\frac{w}{2}-\Delta ,$$ (2)

*a* = 3.0 × 10^12^ V/m^2^, and the charge-depletion width *Δ* is 0.5 μm [15]. The confining potential and magnetic flux density produce an electron current that circulates around the periphery of the device in a counterclockwise direction. This is equivalent to an edge current of positive charges in a clockwise direction.

The charge-redistribution potential *V*_r_(*y*′) is

$$V_{r}\left(y\prime \right)=-\frac{I_{r}R_{H}}{2}G\ln\left|\frac{y\prime +w/2}{y\prime -w/2}\right|,$$ (3)

where *I*_r_ is that part of the total current *I*_SD_ due to the charge-redistribution potential. The value of the geometry factor *G* is 0.147 [12].

The total potential *V*_t_(*y*′) is

$$V_{t}\left(y\prime \right)=V_{c}\left(y\prime \right)+V_{r}\left(y\prime \right).$$ (4)

The electric field equations are

$$E_{c}\left(y\prime \right)=2a\left(y\prime -\lambda \right)\;\text{for}\;\lambda \leq y\prime \leq \frac{w}{2}$$ (5a)

$$E_{c}\left(y\prime \right)=0\;\text{for}\;-\lambda <y\prime <\lambda$$ (5b)

$$E_{c}\left(y\prime \right)=2a\left(y\prime -\lambda \right)\;\text{for}\;-\frac{w}{2}\leq -y\prime \leq -\lambda ,$$ (5c)

and

$$E_{r}\left(y\prime \right)=\frac{I_{r}R_{H}}{2}G\frac{w}{\left[{\left(w/2\right)}^{2}-{\left(y\prime \right)}^{2}\right]},$$ (6)

and

$$E_{t}\left(y\prime \right)=E_{c}\left(y\prime \right)+E_{r}\left(y\prime \right).$$ (7)

The total current density *J*_t_(*y*′) is

$$J_{t}\left(y\prime \right)=J_{c}\left(y\prime \right)+J_{r}\left(y\prime \right)=\frac{1}{R_{H}}E_{t}\left(y\prime \right),$$ (8)

and the applied current *I*_SD_ is

$$I_{\mathrm{SD}}=\int_{y_{\min}}^{y_{\max}}J_{t}\left(y\prime \right)dy\prime .$$ (9)

**Fig. 2:**
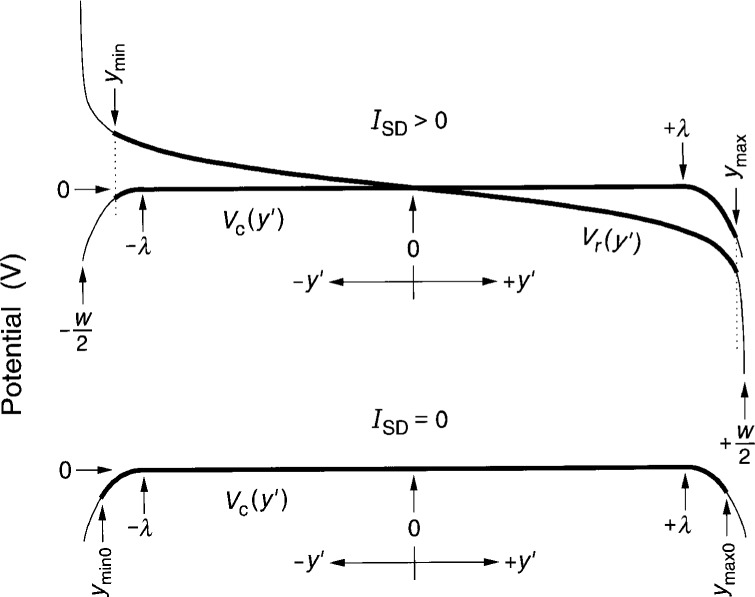
Schematic drawings of the confining potentials *V*_c_(*y*′) and the charge-redistribution potential *V*_r_(*y*′) across the device width for *I*_SD_ = 0 and *I*_SD_ > 0, where *λ* and –*λ* are the locations where the confining potentials begin to deviate from zero. The thick lines between *y*_min0_ and *y*_max0_ for *I*_SD_ = 0, and between *y*_min_ and *y*_max_ for *I*_SD_ > 0 are the regions where the 2DEG electrons are conducting. For clarity, the confining potentials extend far into the device interior, as do the values of *y*_min0_, *y*_max0_, *y*_min_ and *y*_max_.

Figure 2 shows schematic diagrams of *V*_c_(*y*′) and *V*_r_(*y*′) for *I*_SD_ = 0 and *I*_SD_ > 0. For clarity, the confining potentials *V*_c_(*y*′) have been stretched out over wide regions of the device. The thick curves are those parts of the potentials where electrons of the 2DEG occupy Landau states and contribute to the current. Refer to Figs. 7 and 9 of Ref. [12] for actual plots of *V*_t_(*y*′) and *J*_t_(*y*′) versus *y*′ at *I*_SD_ = 25 μA.

*I*_SD_ is an alternating current for impedance measurements. The filled states and thick lines of Fig. 2 shift to the right with increasing current. When *I*_SD_ < 0, *V*_r_(*y*′) has the opposite sign, *V*_c_(*y*′) does not change sign, and the occupied states shift to the left. We will choose an instant in time for the calculations when *I*_SD_ has the root-mean-square (rms) value and is in the negative *x* direction, as shown in Fig. 1.

### 2.2 Parameters Used in the Calculations

A typical ac quantized Hall resistance device is 400 μm wide, has a rms current of about 25 μA, and operates on the *i* = 2 plateau. The following set of values found by Cage and Lavine [12] can therefore be used in our calculations: *I*_SD_ = 25 μA, *R*_H_ = 12 906.4035 Ω, *V*_H_ = *R*_H_*I*_SD_ = 0.3227 V, *B* = 12.3 T, *w* = 400 μm, *a* = 3.0 × 10^12^ V/m^2^, *Δ* = 0.5 μm, *λ* = 199.500 μm, *I*_r_ = 24.74 μA, *G* = 0.147, *y*_max_ = 199.564 μm, *y*_min_ = −199.554 μm, *V*_c_(*y*_max_) = − 0.0122 V, *V*_c_(*y*_min_) = − 0.0088 V, *V*_r_(*y*_max_) = −0.1599 V, *V*_r_(*y*_min_) = 0.1594 V, *E*_c_(*y*_max_) = 3.821 × 10^5^ V/m, *E*_c_(*y*_min_) = −3.255 × 10^5^ V/m, *E*_r_(*y*_max_) = 5.380 × 10^4^ V/m, *E*_r_(0) = 2.345 × 10^2^ V/m, and *E*_r_(*y*_min_) = 5.266 × 10^4^ V/m. Their device length *L_x_* was 4.6 mm. The reduced mass m* of the electron in GaAs is 0.068 times the free electron mass, or 6.194 × 10^−32^ kg.

We will also use the values *y*_max0_ = *− y*_min0_ = 199.559 μm, and *E*_c_(*y*_max0_) = − *E*_c_(*y*_min0_) = 3.54 × 10^5^ V/m from Cage and Levine [12] when *I*_SD_ = 0 μA.

## 3. Internal Capacitance

The quantum Hall voltage *V*_H_(*i*) = *R*_H_(*i*)*I*_SD_ arises because the conducting electrons are shifted slightly towards one side of the device such that the Lorentz force *e****v*** × ***B*** equals the Coulomb repulsive force − *e****E***_t_ everywhere within the 2DEG [16], where ***v*** is the velocity of a conducting electron located at coordinates *x*′, *y*′. This shift in position with applied current *I*_SD_ causes a deviation, − *eδ* σ(*y*′), or charge-redistribution of the electrons in the 2DEG from the average electron surface charge density *en*_S_, where −*δ*σ is the surface density deviation at coordinates *x*′, *y*′ and *n*_S_ = *i(eB*/*h*) is the average surface number density, e.g., 5.94 × 10^11^/cm^2^ for the *i* = 2 plateau at 12.3 T. The charge-redistribution gives rise to separated charges and an internal capacitance across the device width.

### 3.1 Calculations

There is an excess of electrons, with total charge −*Q*, on the right hand side of Fig. 1 and a depletion of electrons, with total charge +*Q*, on the left hand side, where

$$-Q=\int_{0}^{L_{x}}\int_{0}^{w/2}e\delta \sigma \left(y\prime \right)dy\prime dx\prime ,$$ (10a)

$$Q=\int_{0}^{L_{x}}\int_{-w/2}^{0}e\delta \sigma \left(y\prime \right)dy\prime dx\prime ,$$ (10b)

and *L_x_* is the length of the device (neglecting corner effects). Appendix A of Ref. [12] showed that the surface charge-redistribution is

$$\delta \sigma \left(y\prime \right)=\frac{im*}{hB}\frac{d^{2}}{d{y\prime }^{2}}V_{t}\left(y\prime \right),$$ (11)

where m* is the reduced mass of the electron in GaAs (0.068 times the free electron mass).

Note that only those parts of the total potential *V*_t_(*y*′) which *change* with applied current *I*_SD_ should be used in Eqs. (10) and (11) to calculate charge separations within a capacitor. Thus the entire charge-redistribution potential *V*_r_(*y*′) contributes to Eq. (11), and the limits of integration in Eqs. (10) are between *y*_min_ and 0 and between 0 and *y*_max_. However, only those parts of the confining potential *V*_c_(*y*′) which *differ* from the *I*_SD_ = 0 μA case contribute, i.e., the parts between *y*_min0_ and *y*_min_ and between *y*_max0_ and *y*_max_. The parts of *V*_c_(*y*′) between *y*_min_ and *y*_max0_ do *not* contribute because there is no difference from the *I*_SD_ = 0 μA case of Fig. 2.

The total charges − *Q* and + *Q*, defined by Eqs. (10) and (11), are

$$\begin{array}{l} -Q=-e\left(\frac{im*}{hB}\right)\\ \times L_{x}\{[E_{c}\left(y_{\max}\right)-E_{c}\left(y_{\max0}\right)]+[E_{r}\left(y_{\max}\right)-E_{r}\left(0\right)]\} \end{array}$$ (12a)

$$\begin{array}{l} Q=e\left(\frac{im*}{hB}\right)\\ \times L_{x}\{[E_{c}\left(y_{\min}\right)-E_{c}\left(y_{\min0}\right)]+[E_{r}\left(y_{\min}\right)-E_{r}\left(0\right)]\}. \end{array}$$ (12b)

Using the values from Sec. 2.2, Eqs. (12) predict values of −*Q* and +*Q* of −9.36 × 10^−16^ C and 9.23 × 10^−16^ C, respectively for the right and left hand sides of the 4.6 mm long device at *I*_SD_ = 25 μA and *B* = 12.3 T. The confining potential contributes 36 % to the charge *Q*.

There is a 0.7 % difference in the values of *Q* between the two sides of the device, but that does not violate charge conservation. The solutions of Cage and Lavine [12] are self-consistent because charges on both sides of the device are transferred between donor sites in the AlGaAs layer and the 2DEG in the GaAs layer in order to maintain zero net charge within the device volume. It is this charge transfer *between* layers that gives rise to the charge separation +*Q* and −*Q* in the 2DEG.

The separated total charges +*Q* and −*Q* within the 2DEG generate a potential difference *V*_H_ across the device width, producing an internal capacitance *C*_H_ across the device, where *C*_H_ is defined as

$$Q=C_{H}V_{H},$$ (13)

or

$$\begin{array}{c} C_{H}=\left(\frac{e}{V_{H}}\right)\left(\frac{im*}{hB}\right)\\ \times L_{x}\{[E_{c}\left(y_{\max}\right)-E_{c}\left(y_{\max0}\right)]-[E_{r}\left(y_{\max}\right)-E_{r}\left(0\right)]\}. \end{array}$$ (14)

Using the electric field values listed in Sec. 2.2, the capacitance per length is 0.63 pF/m for 400 μm wide devices. The capacitance *C*_H_ is thus about 0.0029 pF for the 4.6 mm long GaAs/AlGaAs device of Ref. [12], and about 0.0014 pF for the widely-used, 2.2 mm long and 400 μm wide, GaAs/AlGaAs BIPM/EUROMET devices [17].

Two frequencies *f* often used in ac quantized Hall resistance measurements are 1233 Hz and 1592 Hz. These correspond to angular frequencies ω = 2*πf* of 7747 rad/s and 10 000 rad/s, respectively, or about 10^4^ rad/s. The impedance *j/(ωC*_H_) due to the Hall capacitance *C*_H_ is about 7×10^10^ for BIPM/EUROMET devices, yielding a correction to *R*_H_ of 2 × 10^−7^ *R*_H_ for the *i* = 2 plateau. This quadrature component correction to the Hall impedance is small, but not insignificant.

We thus predict an internal capacitance *C*_H_ *across* the device width in parallel with the Hall resistance *R*_H_. There is no internal capacitance C*_x_* along the device length within the nearly dissipationless conduction region because the potential difference between *V_x_* probes is negligible and there is no charge separation along the sample length. A potential difference slightly greater than *V*_H_ does occur between the source and drain due to the current entering and exiting at opposite device corners, but this voltage arises from resistive heating rather than charge separation. So the impedance due to *C_x_* along the device length is negligible compared with *R_x_* = *V_x_*/*I*_SD_.

### 3.2 Line Charges Approximation

The total charges +*Q* and −*Q* are concentrated near the positions *y*_min_ and *y*_max_ because that is where the surface charge-redistributions *eδσ* (*y*′) are largest. (See Fig. 11 of Ref. [12] for an example of the charge-redistributions at *I*_SD_ = 215 μA.) One can closely approximate the charge-redistributions as two line charges +*Q*/*L_x_* and −*Q*/*L_x_*, with radii *ρ* that are about one-half the probability distribution thickness of the 2DEG [2], and are separated by the device width *w*. It can be shown using Gauss’s law, *ε* ∫ ***E*** · d***S*** = *Q*, and the definitions of potential, *V* = −∫ ***E*** · d***l***, and capacitance, *C* = *Q*/*V*, that the capacitance between two line charges of radii *ρ* is *πεL_x_*/ln[(*w*− *ρ*)/*ρ*], where *ε* is the permittivity of GaAs (which is 13.1 times larger than the permittivity of a vacuum), *ρ* is about 2.5 nm, d***S*** is an elemental area of the integration surface, and d***l*** is an incremental length along the integration path. The capacitance between two line charges is 48 times larger than that predicted by Eq. (14) for the 2DEG.

Two line charges are not a good approximation of a quantum Hall device, however, because it neglects the large screening effects in the nearby AlGaAs layer of the heterostructure. Charges of opposite sign to the line charges occur in the two regions of the AlGaAs layer near the device sides because the 2DEG arises from electrons tunneling from the AlGaAs layer. There are really four line charges to consider, with charge densities ±*Q*/*L_x_* and ∓*Q*/*L_x_* located on either side of the device. This greatly reduces the electric fields within the 2DEG, and thereby decreases the capacitance across the device. The capacitance predicted by Eq. (14) accounts for these screening effects because it uses electric fields derived from experimental results.

## 4. Kinetic Inductance

The conducting electrons have an inertial mass m*, which gives rise to a kinetic inductance [18] when the current is reversed. Seppa, Satrapinski, Varpula, and Saari [11] predict that this yields a large impedance along the device length. We will examine their results in Sec. 4.2 after deriving our equations for the kinetic inductance.

### 4.1 Calculations

The conducting electrons of Figs. 1 and 2 have a velocity *v_x_* (*y*′) = *E_y_* (*y*′)/*B_z_* = *E*_t_(*y*′)/*B_z_* and a kinetic energy $\frac{1}{2}m*v_{x}^{2}(y\prime )$. Neglecting corner effects, the total kinetic energy *K* within the 2DEG of a device of length *L_x_* is

$$K=\frac{1}{2}\int_{0}^{L_{x}}\int_{-w/2}^{w/2}m*v_{x}^{2}\left(y\prime \right)n_{s}dy\prime dx\prime .$$ (15)

Noting that *J*_t_(*y*′) = *J_x_* (*y*′) = *n*_S_*ev_x_* (*y*′), *n*_S_ = *i*(*eB/h*), *R*_H_ = *h*/(*e*^2^*i*), and *J*_t_(*y*′) = *E*_t_(*y*′)/*R*_H_, Eq. (15) can be rewritten as

$$\begin{array}{c} K=\frac{1}{2}\left[\left(\frac{m*R_{H}}{eBI_{\mathrm{SD}}^{2}}\right)L_{x}\int_{-w/2}^{w/2}J_{t}^{2}\left(y\prime \right)dy\prime \right]I_{\mathrm{SD}}^{2}\\ =\frac{1}{2}L_{k}I_{\mathrm{SD}}^{2}, \end{array}$$ (16)

where the kinetic inductance *L*_k_ is

$$\begin{array}{l} L_{k}=\left(\frac{m*R_{H}}{eBI_{\mathrm{SD}}^{2}}\right)L_{x}\int_{-w/2}^{w/2}{\left[J_{t}\left(y\prime \right)\right]}^{2}dy\prime \\ =\left(\frac{m*}{eBR_{H}I_{\mathrm{SD}}^{2}}\right)L_{x}\int_{-w/2}^{w/2}[E_{r}\left(y\prime \right)+E_{c}{\left(y\prime \right)}^{2}]dy\prime . \end{array}$$ (17)

Please note that *L*_k_ is the kinetic inductance and *L_x_* is the device length.

Eqs. (5) and (6) can be used in Eq. (17), remembering that only those parts of the electric fields which *change* with applied current *I*_SD_ should be included. This means all of the charge-redistribution electric field *E*_r_(*y*′), integrated between the limits *y*_min_ and *y*_max_, but only those parts of the confining field *E*_c_(*y*′) which differ from the *I*_SD_ = 0 μA case, i.e., those parts between *y*_min0_ and *y*_min_ and between *y*_max0_ and *y*_max_. The parts between *y*_min_ and −*λ* and between *λ* and *y*_max0_ do not contribute to *L*_k_ because they result from an internal dc current which circulates around the device periphery and is independent of *I*_SD_.

The integrals of Eq. (17) are analytic, and have the solution

$$L_{k}=A\left(B+C+D+E\right)$$ (18a)

where

$$A=\left(\frac{m*}{eBR_{H}I_{\mathrm{SD}}^{2}}\right)L_{x}$$ (18b)

$$\begin{array}{l} B=\frac{I_{r}R_{H}G}{w}\\ \;\times [y_{\max}E_{r}\left(y_{\max}\right)-y_{\min}E_{r}\left(y_{\min}\right)-V_{r}\left(y_{\max}\right)+V_{r}\left(y_{\min}\right)] \end{array}$$ (18c)

$$\begin{array}{l} C=4a^{2}\\ \;\times \left[\frac{1}{3}\left(y_{\max}^{3}+y_{\min}^{3}\right)-\lambda \left(y_{\max}^{3}-y_{\min}^{3}\right)+\lambda^{2}\left(y_{\max}+y_{\min}\right)\right] \end{array}$$ (18d)

$$\begin{array}{l} D=aI_{r}R_{H}Gw\\ \;\times \left\{\ln\frac{\left[{\left(w/2\right)}^{2}-y_{\min0}^{2}\right]}{\left[{\left(w/2\right)}^{2}-y_{\min}^{2}\right]}+\ln\frac{\left[{\left(w/2\right)}^{2}-y_{\max0}^{2}\right]}{\left[{\left(w/2\right)}^{2}-y_{\max}^{2}\right]}\right\} \end{array}$$ (18e)

$$\begin{array}{l} E=4a\lambda \\ \;\times \{[V_{r}\left(y_{\max}\right)-[V_{r}\left(y_{\max0}\right)]-[V_{r}\left(y_{\min}\right)-[V_{r}\left(y_{\min0}\right)]\}. \end{array}$$ (18f)

The kinetic inductance can be evaluated from Eqs. (18) using the values listed in Sec. 2.2, except for term *E* where the values of *V*_r_(*y*′) need to be calculated to more significant figures using Eq. (3). The kinetic inductance per length is about 15 μH/m for 400 μm wide devices with *i* = 2 plateaus at 12.3 T. The terms involving the confining potential (*C*, *D*, and *E*) contribute 33 % of this value. The kinetic inductance *L*_k_ is thus about 0.07 μH for the 4.6 mm long GaAs/AlGaAs device of Ref. [12], and about 0.04 μH for 2.2 mm long GaAs/AlGaAs BIPM/EUROMET devices [17], where the *i* = 2 plateau occurs at about 10 T. These values are for *I*_SD_ = 25 μA. *L*_k_ is somewhat current dependent in this model because *E*_r_(*y*′) scales linearly with *I*_SD_ but *E*_c_(*y*′) does not; so *L*_k_ decreases from 0.07 μH to 0.05 μH between 25 μA and 215 μA. This difference over such a wide current range is small enough to ignore.

The impedance *jωL*_k_ due to the kinetic inductance *L*_k_ is about 0.4 mΩ for BIPM/EUROMET devices at *ω* = 10^4^ rad/s and *I*_SD_ = 25 μA, or only about 3 parts in 10^8^ of *R*_H_ for the *i* = 2 plateau. This out-of-phase impedance component is along the device length, and is comparable in magnitude to the longitudinal resistance *R_x_* = *V_x_*/*I*_SD_.

### 4.2 Uniform Current Density Approximation

Seppa, Satrapinski, Varpula, and Saari [11] considered the case of a *uniform* current density *J*_t_ = *I*_SD_/*w* across the device width *w*. A uniform current density in Eq. (17) yields *L*_k_ = (m*R_H_*L_x_*)/(*eBw*) = (m*L_x_)/(*n*_s_*e*^2^*w*), where *n*_s_ = *i*(*eB*/*h*). For BIPM/EUROMET devices with *i* = 2 plateaus at 10 T we find that *L*_k_ = 0.003 μH for a uniform current density approximation, or about 13 times smaller than the more realistic prediction in Sec. 4.1.

Seppa et al. [11] predicted a much larger value of *L*_k_ = 40 μH for this example than we have because they assumed free electrons with mass *m*_e_, rather than electrons with reduced mass m* in the 2DEG, and a conducting electron number density that was 1000 times smaller than *n*_s_. This last assumption is inconsistent with the requirement that the average surface density is *n*_s_ = *i*(*eB*/*h*) on a quantum Hall plateau.

Using our Eqs. (11), (1), and (3), the deviation −*δσ*(*y*′) in the density of electrons from the average surface density *n*_s_ is

$$-\delta \sigma \left(y\prime \right)=\left(\frac{im*}{hB}\right)\left\{2a+\frac{I_{r}R_{H}G_{wy\prime }}{{\left[{\left(w/2\right)}^{2}-{\left(y\prime \right)}^{2}\right]}^{2}}\right\}.$$ (19)

The largest deviation in the 2DEG occurs at *y*′ = *y*_max_, and has the value −*δσ*(*y*′) = 9.30 × 10^9^/cm^2^ for the *i* = 2 plateau at 12.3 T and *I*_SD_ = 25 μA. This is only 1.6 % of *n*_s_ = 5.94 × 10^11^/cm^2^, and satisfies the further requirement in the model of Cage and Lavine [12] that the charge density varies slowly across the device width.

## 5. Magnetic Inductance

Determining the magnetic inductance *L*_m_ of a quantum Hall effect device is not quite as straightforward as determining the Hall capacitance *C*_H_ or the kinetic inductance *L*_k_. The device can be treated as an isolated object when calculating values for *C*_H_ and *L*_k_. The magnetic inductance, however, can only be evaluated when the device is part of a complete current-carrying circuit. Therefore, *L*_m_ depends on the circuit geometry.

We chose a geometry in which the device is represented as a current sheet, with a return wire located below the middle of the sheet because this geometry approximates the source-drain leads of a typical sample probe. The integral equations for *L*_m_ have analytic solutions for this geometry, and values of *L*_m_ can be compared with the values for two parallel wires carrying currents in opposite directions.

We consider only the magnetic inductance *outside* the sheet and the wire. The self-inductance per length *inside* a long, nonpermeable, cylindrically-shaped wire is *μ*_0_/(8*π*) [19], where *μ*_0_ = 4*π* × 10^−7^ H/m is the permeability of free space.

### 5.1 Calculations

**Fig. 3:**
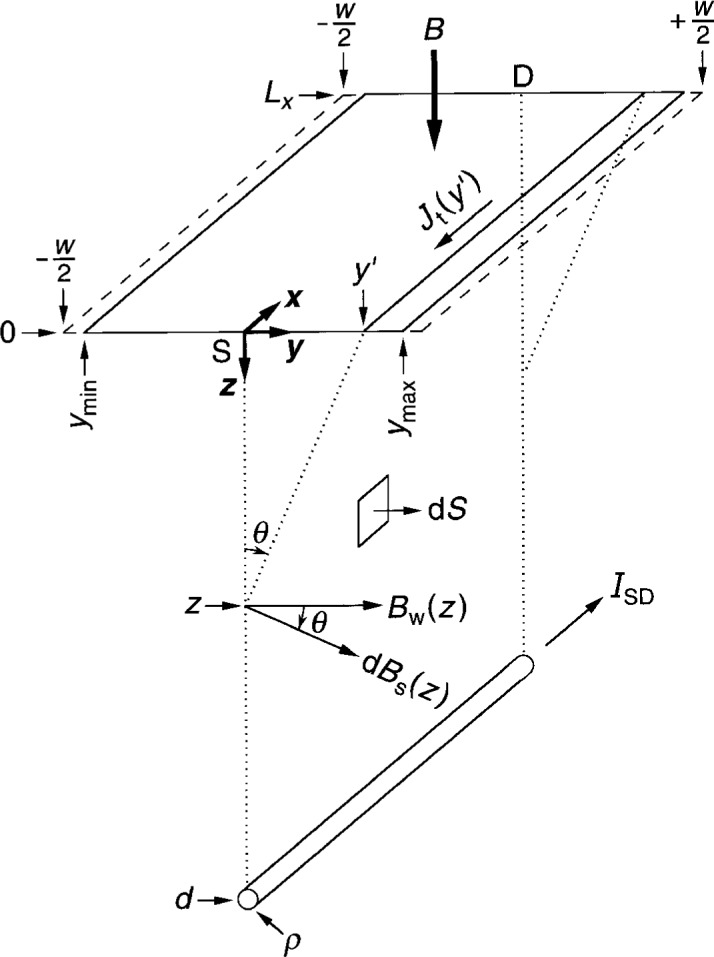
A sketch of the 2DEG conducting sheet and a parallel current return wire of radius *ρ*, located a distance *d* from the middle of the sheet. *B*_s_(*z*) and *B*_w_(*z*) are magnetic flux densities generated by the conducting sheet and wire. d*S* = d*x*d*z* is an elemental area in the *x* –*z* plane located between the conducting sheet and the wire.

Figure 3 shows the circuit geometry. The current-carrying 2DEG sheet and parallel return wire each extend to ±∞ along the *x* axis. The current density is *J*_t_(*y*′) = *E*_t_(*y*′)/*R*_H_ within the conducting sheet, where *E*_t_(*y*′) is given by Eqs. (5) and (6). The return wire has a radius *ρ*, and is separated from the sheet by a distance *d* from the origin. The wire carries a current

$$I_{\mathrm{SD}}=\int_{-w/2}^{w/2}J_{t}\left(y\prime \right)dy\prime$$ (20)

in the opposite direction to that in the sheet.

The magnetic flux *ϕ*_m_ and magnetic inductance *L*_m_ are defined by

$$\phi_{m}=\int B_{m}\cdot dS=\oint A\cdot dl=L_{m}I_{\mathrm{SD}},$$ (21)

where ***B***_m_ is the magnetic flux density generated by both the conducting sheet and the return wire, d***S*** is an elemental area of the enclosed current-carrying circuit, ***A*** is the vector potential, d***l*** is an incremental length along the path around the circuit just outside the conductors, and *I*_SD_ is the applied current [19].

We chose d***S*** to be located in Fig. 3 in the *x*–*z* plane between the conducting sheet and the wire at *y* = 0; so d***S*** = d*x*d*z*. Therefore, only the *y*-components of ***B***_m_ perpendicular to d***S*** are needed to evaluate the surface integral in Eq. (21). These *y*-components of ***B***_m_ are

$$B_{m}\left(z\right)=B_{w}\left(z\right)+B_{s}\left(z\right)\cos\theta =B_{w}\left(z\right)+B_{r}\left(z\right)+B_{c}\left(z\right),$$ (22)

where *B*_w_(*z*) is due to the return wire. *B*_s_(*z*)cos*θ* is due to the conducting sheet, and is composed of charge-redistribution and confining parts *B*_r_(*z*) and *B*_c_(*z*), respectively. *B*_w_(*z*) is easily obtained from Ampere’s law ∫***B***_w_ · d***l*** = *μ*_0_*I*_SD_

$$B_{w}\left(z\right)=\frac{\mu_{0}}{2\pi }I_{\mathrm{SD}}\frac{1}{\left(d-z\right)}.$$ (23)

*B*_s_(*z*) is found by considering the conducting sheet as a series of wires carrying currents *J*_t_(*y*′)d*y*′

$$dB_{s}\left(z\right)\cos\theta =\frac{\mu_{0}}{2\pi }J_{t}\left(y\prime \right)\frac{dy\prime }{\sqrt{{\left(y\prime \right)}^{2}+z^{2}}}\frac{z}{\sqrt{{\left(y\prime \right)}^{2}+z^{2}}}$$ (24)

or

$$B_{s}\left(z\right)\cos\theta =B_{r}\left(z\right)+B_{c}\left(z\right)=\frac{\mu_{0}z}{2\pi }\int_{-w/2}^{w/2}\frac{J_{t}\left(y\prime \right)}{\left[{\left(y\prime \right)}^{2}+z^{2}\right]}dy\prime .$$ (25)

This gives

$$\begin{array}{c} B_{r}\left(z\right)=\frac{\mu_{0}}{4\pi }I_{r}Gwz\\ \times \int_{y_{\min}}^{y_{\max}}\frac{1}{\left[{\left(\frac{w}{2}\right)}^{2}-{\left(y\prime \right)}^{2}\right]\left[{\left(y\prime \right)}^{2}+z^{2}\right]}dy\prime , \end{array}$$ (26)

and

$$\begin{array}{c} B_{r}\left(z\right)=\frac{\mu_{0}}{4\pi }I_{r}G\frac{w}{\left[{\left(\frac{w}{2}\right)}^{2}+z^{2}\right]}\\ \times \left\{\arctan\left(\frac{y_{\max}}{z}\right)-\arctan\left(\frac{y_{\min}}{z}\right)\right\}\\ +\frac{\mu_{0}}{4\pi }I_{r}G\frac{z}{\left[{\left(\frac{w}{2}\right)}^{2}+z^{2}\right]}\\ \times \left\{\ln\left[\frac{\frac{w}{2}+y_{\max}}{\frac{w}{2}-y_{\max}}\right]-\ln\left[\frac{\frac{w}{2}+y_{\min}}{\frac{w}{2}-y_{\min}}\right]\right\} \end{array}$$ (27)

for the charge-redistribution term, and

$$\begin{array}{l} B_{c}\left(z\right)=\frac{\mu_{0}a}{\pi R_{H}}z\int_{y_{\min0}}^{y_{\min}}\frac{\left(y\prime +\lambda \right)}{\left[{\left(y\prime \right)}^{2}+z^{2}\right]}dy\prime \\ +\frac{\mu_{0}a}{\pi R_{H}}z\int_{y_{\max0}}^{y_{\max}}\frac{\left(y\prime -\lambda \right)}{\left[{\left(y\prime \right)}^{2}+z^{2}\right]}dy\prime , \end{array}$$ (28)

and

$$\begin{array}{l} B_{c}\left(z\right)=\frac{\mu_{0}a}{\pi R_{H}}\frac{z}{2}\left\{\ln\left[\frac{y_{\max}^{2}+z^{2}}{y_{\max0}^{2}+z^{2}}\right]+\ln\left[\frac{y_{\min}^{2}+z^{2}}{y_{\min0}^{2}+z^{2}}\right]\right\}\\ +\frac{\mu_{0}a}{\pi R_{H}}\lambda \left\{\arctan\left(\frac{y_{\min}}{z}\right)-\arctan\left(\frac{y_{\min0}}{z}\right)\right\}\\ -\frac{\mu_{0}a}{\pi R_{H}}\lambda \left\{\arctan\left(\frac{y_{\max}}{z}\right)-\arctan\left(\frac{y_{\max0}}{z}\right)\right\} \end{array}$$ (29)

for the confining term. The integrals in Eq. (28) extend only between *y*_min0_ and *y*_min_ and between *y*_max0_ and *y*_max_ because we are interested in the parts of *B*_c_(*z*) that *change* when *I*_SD_ changes. The parts between *y*_min_ and −*λ* and between *λ* and *y*_max0_ provide a constant magnetic flux density that does not contribute to the magnetic inductance.

If the quantum mechanical probability distribution of the 2DEG extends over a distance 2*ρ*, if the device length is *L_x_*, and if we neglect the corner effects, then Eqs. (21) and (22) yield

$$\begin{array}{c} L_{m}=L_{W}+L_{r}+L_{c}\\ =\frac{1}{I_{\mathrm{SD}}}\int_{0}^{L_{x}}\int_{\rho }^{d-\rho }[B_{W}\left(z\right)+B_{r}\left(z\right)+B_{c}\left(z\right)]dzdx. \end{array}$$ (30)

Using Eqs. (23), (27), and (29) in Eq. (30), we find that

$$L_{W}=\frac{\mu_{0}}{2\pi }L_{x}\ln\left[\frac{d-\rho }{\rho }\right],$$ (31a)

and

$$\begin{array}{c} L_{r}\approx \frac{\mu_{0}}{2\pi }\frac{I_{r}}{I_{\mathrm{SD}}}GL_{x}\left\{{\left[\arctan\left(\frac{w}{2\rho }\right)\right]}^{2}-{\left[\arctan\left(\frac{w}{2\left(d-\rho \right)}\right)\right]}^{2}\right\}\\ +\frac{\mu_{0}}{8\pi }\frac{I_{r}}{I_{\mathrm{SD}}}GL_{x}\left\{\ln\left[\frac{\frac{w}{2}+y_{\max}}{\frac{w}{2}-y_{\max}}\right]-\ln\left[\frac{\frac{w}{2}+y_{\min}}{\frac{w}{2}-y_{\min}}\right]\right\}\\ \times \left\{\ln\left[\frac{(\frac{w}{2}{)}^{2}+{\left(d-\rho \right)}^{2}}{(\frac{w}{2}{)}^{2}+\rho^{2}}\right]\right\}, \end{array}$$ (31b)

and

$$\begin{array}{c} L_{c}=\frac{\mu_{0}a}{4\pi R_{H}I_{\mathrm{SD}}}L_{x}\left\{y_{\max}^{2}\ln\left[\frac{y_{\max}^{2}+{\left(d-\rho \right)}^{2}}{y_{\max}^{2}+\rho^{2}}\right]-y_{\max0}^{2}\ln\left[\frac{y_{\max0}^{2}+{\left(d-\rho \right)}^{2}}{y_{\max}^{2}+\rho^{2}}\right]\right\}\\ +\frac{\mu_{0}a}{4\pi R_{H}I_{\mathrm{SD}}}L_{x}\left\{{\left(d-\rho \right)}^{2}\ln\left[\frac{y_{\max}^{2}+{\left(d-\rho \right)}^{2}}{y_{\max0}^{2}+{\left(d-\rho \right)}^{2}}\right]-\rho^{2}\ln\left[\frac{y_{\max}^{2}+\rho^{2}}{y_{\max0}^{2}+\rho^{2}}\right]\right\}+\frac{\mu_{0}a}{4\pi R_{H}I_{\mathrm{SD}}}\\ \times L_{x}\left\{y_{\min}^{2}\ln\left[\frac{y_{\min}^{2}+{\left(d-\rho \right)}^{2}}{y_{\min}^{2}+\rho^{2}}\right]-y_{\min0}^{2}\ln\left[\frac{y_{\min0}^{2}+{\left(d-\rho \right)}^{2}}{y_{\min0}^{2}+\rho^{2}}\right]\right\}+\frac{\mu_{0}a}{4\pi R_{H}I_{\mathrm{SD}}}L_{x}\{{\left(d-\rho \right)}^{2}\ln\left[\frac{y_{\min}^{2}+{\left(d-\rho \right)}^{2}}{y_{\min0}^{2}+{\left(d-\rho \right)}^{2}}\right]\\ -\rho^{2}\ln\left[\frac{y_{\min}^{2}+\rho^{2}}{y_{\min0}^{2}+\rho^{2}}\right]\}+\frac{\mu_{0}a}{4\pi R_{H}I_{\mathrm{SD}}}\lambda L_{x}\left\{y_{\min}\ln\left[\frac{y_{\min}^{2}+{\left(d-\rho \right)}^{2}}{y_{\min}^{2}+\rho^{2}}\right]-y_{\min0}\ln\left[\frac{y_{\min0}^{2}+{\left(d-\rho \right)}^{2}}{y_{\min0}^{2}+\rho^{2}}\right]\right\}\\ -\frac{\mu_{0}a}{2\pi R_{H}I_{\mathrm{SD}}}\lambda L_{x}\left\{y_{\max}\ln\left[\frac{y_{\max}^{2}+{\left(d-\rho \right)}^{2}}{y_{\max}^{2}+\rho^{2}}\right]-y_{\max0}\ln\left[\frac{y_{\max0}^{2}+{\left(d-\rho \right)}^{2}}{y_{\max0}^{2}+\rho^{2}}\right]\right\}\\ +\frac{\mu_{0}a}{\pi R_{H}I_{\mathrm{SD}}}\lambda L_{x}\left\{\left(d-\rho \right)\left[\arctan\left(\frac{y_{\min}}{d-\rho }\right)-\arctan\left(\frac{y_{\min0}}{d-\rho }\right)\right]\right\}-\frac{\mu_{0}a}{\pi R_{H}I_{\mathrm{SD}}}\lambda L_{x}\{\left(\rho \right)\left[\arctan\left(\frac{y_{\min}}{\rho }\right)\right]\\ -\arctan\left(\frac{y_{\min0}}{\rho }\right)]\}-\frac{\mu_{0}a}{\pi R_{H}I_{\mathrm{SD}}}\lambda L_{x}\left\{\left(d-\rho \right)\left[\arctan\left(\frac{y_{\max}}{d-\rho }\right)-\arctan\left(\frac{y_{\max0}}{d-\rho }\right)\right]\right\}\\ +\frac{\mu_{0}a}{\pi R_{H}I_{\mathrm{SD}}}\lambda L_{x}\left\{\left(\rho \right)\left[\arctan\left(\frac{y_{\max}}{\rho }\right)-\arctan\left(\frac{y_{\max0}}{\rho }\right)\right]\right\}. \end{array}$$ (31c)

We made the approximation *y*_max_ ≈ − *y*_min_ ≈ *w*/2 in the arctan terms of Eq. (27) in order to obtain analytic solutions for the arctan terms of Eq. (31b). These approximate analytic solutions agree with complete numerical integrations to within 3 parts in 10^4^.

### 5.2 Comparison with Two Parallel Wires

If the conducting sheet of Fig. 3 is replaced with a wire of radius *ρ* located at the origin, and this wire has an applied current *I*_SD_ of positive charges flowing in the negative *x* direction, then the magnetic inductance *L*_loop_ of the current loop is

$$\begin{array}{l} L_{\mathrm{loop}}=\frac{\mu_{0}}{2\pi }L_{x}\int_{\rho }^{d-\rho }\left[\frac{1}{z}+\frac{1}{d-z}\right]dz\\ =\frac{\mu_{0}}{\pi }L_{x}\ln\left[\frac{d-\rho }{\rho }\right]=2L_{W}. \end{array}$$ (32)

**Fig. 4:**
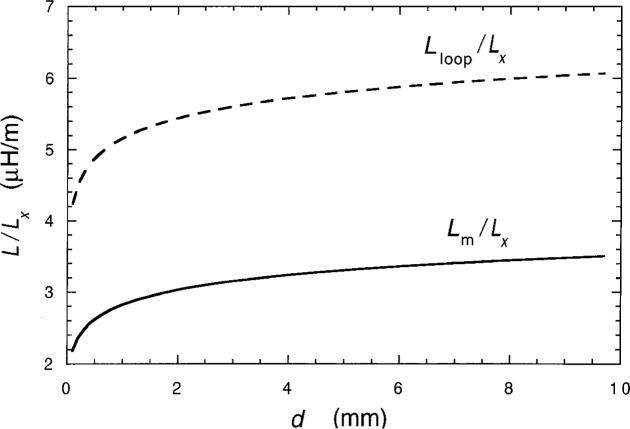
A comparison of the magnetic inductances per length for a conducting sheet and return wire, *L*_m_/*L_x_*, and for a conducting wire and return wire, *L*_loop_/*L_x_*, when using separation distances *d* between 0.1 mm and 10 mm, the parameters listed in Sec. 2.2 for *I*_SD_ = 25 μA, and assuming that the wire radius *ρ* is 2.5 nm.

The magnetic inductances per length, *L*_m_/*L_x_* and *L*_loop_/*L_x_*, are compared in Fig. 4 for distances *d* between 0.1 mm and 10 mm, assuming that *ρ* = 2.5 nm and using the parameters listed in Sec. 2.2 for *I*_SD_ = 25 μA. *L*_m_ of the current sheet and return wire configuration is always less than the value of *L*_loop_ for the two parallel wires configuration. Therefore, an over-estimate of the magnetic inductance of a quantum Hall device can be made for a particular experimental arrangement by assuming the device is replaced with a wire of radius *ρ* and length *L_x_*.

## 6. Conclusions

We predict that the capacitance per length is about 0.63 pF/m for 400 μm wide devices on the *i* = 2 plateau, and thus that the internal capacitance *C*_H_ across the device width is about 0.0014 pF for 2.2 mm long BIPM/EUROMET devices. This gives an out-of-phase (quadrature) impedance of about 7 × 10^10^ Ω, which is a correction of about 2 parts in 10^7^ of the in-phase value of *R*_H_ for BIPM/EUROMET devices at *ω* = 10^4^ rad/s. This out-of-phase impedance component correction is small, but not insignificant.

The kinetic inductance per length is about 15 μH/m for 400 μm wide devices with *i* = 2 plateaus at 12.3 T, and about 18 μH/m for devices with *i* = 2 plateaus at 10 T. The kinetic inductance *L*_k_ is thus about 0.04 μH for 2.2 mm long BIPM/EUROMET devices. The quadrature impedance due to the kinetic inductance is along the device length. It has a value of about 0.4 mΩ for BIPM/EUROMET devices at *ω* = 10^4^ rad/s, or only about a 3 parts in 10^8^ out-of-phase correction to the value of *R*_H_ for the *i* = 2 plateau. The kinetic inductance out-of-phase impedance is comparable in value to the in-phase longitudinal resistance *R_x_*.

The magnetic inductance along the device length can only be calculated for known configurations. Thus its value depends on the experimental arrangement. We have shown, however, that an upper-limit estimate of the value can be obtained by replacing the device with a wire of radius *ρ* and length *L_x_*.

The internal capacitances, kinetic inductances, and magnetic inductances calculated here result from the quantum Hall effect device itself (although the magnetic inductances necessarily included the effects of a return wire placed in a particular geometrical arrangement). There are also capacitances, inductances, and resistances associated with external lead connections to the device, with electrical shields placed around the device, and with contact resistances to the 2DEG. The impedances of these additional circuit elements were not considered here, but they must also be accounted for if the impedance standard is to have the intrinsic in-phase value of *R*_H_(*i*).
